# Supplementary material for: Contribution of the tobamovirus resistance gene Tm-1 to control of tomato brown rugose fruit virus (ToBRFV) resistance in tomato
Source: PLoS Genet. 2025 May 23;21(5):e1011725. doi: 10.1371/journal.pgen.1011725 (PMC12140429; doi:10.1371/journal.pgen.1011725)
Supplement: S1 Fig — (DOCX) [file pgen.1011725.s001.docx]

**S1 Fig**. *Tm-1* coding sequence nucleotide alignment

***Tm-1 GCR237*** 1 ATGGCAACTGCACAGAGTAATTCTCCTCGAGTTTTCTGTATCGGAACAGCTGATACAAAATTCGACGAGC

***Tm-1 VC554 1st*** 1 ATGGCAACTGCACAGAGTAATTCTCCTCGAGTTTTCTGTATCGGAACAGCTGATACAAAATTCGACGAGC

***tm-1 GCR26***  1 ATGGCAACTGCACAGAGTAATTCTCCTCGAGTTTTCTGTATCGGAACAGCTGATACTAAATTCGACGAGC

***tm-1 Moneymaker*** 1 ATGGCAACTGCACAGAGTAATTCTCCTCGAGTTTTCTGTATCGGAACAGCTGATACTAAATTCGACGAGC

***tm-1 VC532***  1 ATGGCAACTGCACAGAGTAATTCTCCTCGAGTTTTCTGTATCGGAACAGCTGATACTAAATTCGACGAGC

***Tm-1 VC554 2nd***  1 ATGGCAAGTGCACAGAGTAATTCTCCTCGAGTTTTCTGTATTGGAACAGCTGATACTAAATTCGACGAGC

***Tm-1 GCR237*** 71 TTCGTTTCCTCTCCGAGCATGTGAGATCCAGTCTTAACAGCTTCTCCAATAAATCCTCATTCAAGGTAGG

***Tm-1 VC554 1st*** 71 TTCGTTTCCTCTCCGAGCATGTGAGATCCAGTCTTAACAGCTTCTCCAATAAATCCTCATTCAAGGTAGG

***tm-1 GCR26***  71 TTCGTTTCCTCTCCGAGCATGTGAGATCCAGTCTTAACAGCTTCTCCAATAAATCCTCATTCAAGGTAGG

***tm-1 Moneymaker*** 71 TTCGTTTCCTCTCCGAGCATGTGAGATCCAGTCTTAACAGCTTCTCCAATAAATCCTCATTCAAGGTAGG

***tm-1 VC532***  71 TTCGTTTCCTCTCCGAGCATGTGAGATCCAGTCTTAACAGCTTCTCCAATAAATCCTCATTCAAGGTAGG

***Tm-1 VC554 2nd***  71 TTCGTTTCCTCTCCCAATATGTGAGATCCAGTCTTAACAGCTTCTCCAATAAATCCTCATTCAAGGTCGG

***Tm-1 GCR237*** 141 AGTGACAGTTGTTGATGTCTCAACCAGCTGGAAGGAGACAAATAGTTGTGCTGATTTTGATTTTGTACCG

***Tm-1 VC554 1st*** 141 AGTGACAGTTGTTGATGTCTCAACCAGCTGGAAGGAGACAAATAGTTGTGCTGATTTTGATTTTGTACCG

***tm-1 GCR26***  141 AGTGACAGTTGTTGATGTCTCAACCAGCCGGAAGGAGACAAATAGTTGTGCTGATTTTGATTTTGTACCG

***tm-1 Moneymaker*** 141 AGTGACAGTTGTTGATGTCTCAACCAGCCGGAAGGAGACAAATAGTTGTGCTGATTTTGATTTTGTACCG

***tm-1 VC532***  141 AGTGACAGTTGTTGATGTCTCAACCAGCCGGAAGGAGACAAATAGTTGTGCTGATTTTGATTTTGTACCG

***Tm-1 VC554 2nd***  141 AGTCACAGTTGTTGATGTCTCAACCAGCCTAAAGGAGACAAATGGTTGTGCTGATTTTGATTTTGTGCCG

***Tm-1 GCR237*** 211 AGTAAGGATGTGCTGTCATGCCATACACTAGGGGAAGAAACTATGGGCACGTTTGCAGATATTAGAGGCC

***Tm-1 VC554 1st*** 211 AGTAAGGATGTGCTGTCATGCCATACACTAGGGGAAGAAACTATGGGCACGTTTGCAGATATTAGAGGCC

***tm-1 GCR26***  211 AGTAAGGATGTGCTGTCATGCTATGCACGAGGGGAAGGAACTGTGGGCAGGTTTCCAGATATTAGAGGCC

***tm-1 Moneymaker*** 211 AGTAAGGATGTGCTGTCATGCTATGCACGAGGGGAAGGAACTGTGGGCAGGTTTCCAGATATTAGAGGCC

***tm-1 VC532***  211 AGTAAGGATGTGCTGTCATGCTATGCACGAGGGGAAGGAACTGTGGGCAGGTTTCCAGATATTAGAGGCC

***Tm-1 VC554 2nd***  211 AGGAAGGATGTGCTGTCCTGCTATGCACAAGGGGGAGAATCTGTGGTCCAGCTTCCAGATGATAGAGGCC

***Tm-1 GCR237*** 281 TAGCTATTGCAATCATGAGCAAAGCTCTTGAAACTTTCCTAAGTATAGCTAATGATGAACAGAATCTTGC

***Tm-1 VC554 1st*** 281 TAGCTATTGCAATCATGAGCAAAGCTCTTGAAACTTTCCTAAGTATAGCTAATGATGAACAGAATCTTGC

***tm-1 GCR26***  281 AAGCTATTGCAATCATGAACAAAGCTCTGGAAACTTTCCTAAGTAAAGCTAATGGTGAACAGAATCTTGC

***tm-1 Moneymaker*** 281 AAGCTATTGCAATCATGAACAAAGCTCTGGAAACTTTCCTAAGTAAAGCTAATGGTGAACAGAATCTTGC

***tm-1 VC532***  281 AAGCTATTGCAATCATGAACAAAGCTCTGGAAACTTTCCTAAGTAAAGCTAATGGTGAACAGAATCTTGC

***Tm-1 VC554 2nd***  281 AAGCTATTGCAATCATGAACAAAGCTTTTCAAACTTTCCTAAGCAAAGCTAATGGTGAACAGAATCTTGC

***Tm-1 GCR237*** 351 TGGAGTAATTGGCCTTGGGGGTAGTGGAGGAACATCTCTATTGTCATCTGCCTTCCGATCTCTTCCAATT

***Tm-1 VC554 1st*** 351 TGGAGTAATTGGCCTTGGGGGTAGTGGAGGAACATCTCTATTGTCATCTGCCTTCCGATCTCTTCCAATT

***tm-1 GCR26***  351 TGGAGTGATTGGCCTTGGGGGTAGTGGAGGAACATCTCTATTGTCATCTGCCTTCCGATCTCTTCCAATT

***tm-1 Moneymaker*** 351 TGGAGTGATTGGCCTTGGGGGTAGTGGAGGAACATCTCTATTGTCATCTGCCTTCCGATCTCTTCCAATT

***tm-1 VC532***  351 TGGAGTGATTGGCCTTGGGGGTAGTGGAGGAACATCTCTATTGTCATCTGCCTTCCGATCTCTTCCAATT

***Tm-1 VC554 2nd***  351 TGGAGTGATTGGCCTTGGGGGTAGTGGAGGAACATCTCTATTGTCATCTGCCTTCCGATCTCTTCCAATT

***Tm-1 GCR237*** 421 GGGATCCCAAAAGTTATAATATCTACAGTTGCCAGTGGTCAAACTGAATCTTATATTGGAACATCAGACT

***Tm-1 VC554 1st*** 421 GGGATCCCAAAAGTTATAATATCTACAGTTGCCAGTGGTCAAACTGAATCTTATATTGGAACATCAGACT

***tm-1 GCR26***  421 GGGATCCCAAAAGTTATAATATCTACAGTTGCCAGTGGCCAAACTGAATCTTATATTGGAACATCAGACT

***tm-1 Moneymaker*** 421 GGGATCCCAAAAGTTATAATATCTACAGTTGCCAGTGGCCAAACTGAATCTTATATTGGAACATCAGACT

***tm-1 VC532***  421 GGGATCCCAAAAGTTATAATATCTACAGTTGCCAGTGGCCAAACTGAATCTTATATTGGAACATCAGACT

***Tm-1 VC554 2nd***  421 GGAATCCCAAAAGTTATAATATCTACAGTTGCCAGTGGTCAAACTGAATCTTATATTGGAACATCAGACT

***Tm-1 GCR237*** 491 TGGTATTGTTTCCTTCAGTTGTAGATATTTGTGGGATTAACAATGTCAGTAAGGTTGTTCTATCTAATGC

***Tm-1 VC554 1st*** 491 TGGTATTGTTTCCTTCAGTTGTAGATATTTGTGGGATTAACAATGTCAGTAAGGTTGTTCTATCTAATGC

***tm-1 GCR26***  491 TGGTATTGTTTCCTTCAGTTGTAGATATTTGTGGGATTAACAATGTTAGTAAGGTTGTTCTATCTAATGC

***tm-1 Moneymaker*** 491 TGGTATTGTTTCCTTCAGTTGTAGATATTTGTGGGATTAACAATGTTAGTAAGGTTGTTCTATCTAATGC

***tm-1 VC532***  491 TGGTATTGTTTCCTTCAGTTGTAGATATTTGTGGGATTAACAATGTTAGTAAGGTTGTTCTATCTAATGC

***Tm-1 VC554 2nd***  491 TGGTATTGTTTCCTTCAGTTGTAGATATTTGTGGGATTAACAATGTTAGTAAGGTTATTCTATCTAATGC

***Tm-1 GCR237*** 561 GGGTGCAGCATTTGCTGGAATGGTGATCGGGAGGCTTGAAAGTTCAAAAGAGCATAGCATCACTAATGGA

***Tm-1 VC554 1st*** 561 GGGTGCAGCATTTGCTGGAATGGTGATCGGGAGGCTTGAAAGTTCAAAAGAGCATAGCATCACTAATGGA

***tm-1 GCR26***  561 GGGTGCAGCATTTGCTGGAATGGTGATTGGAAGGCTTGAAAGTTCAAAAGAGCATAGCATCACTAATGGA

***tm-1 Moneymaker*** 561 GGGTGCAGCATTTGCTGGAATGGTGATTGGAAGGCTTGAAAGTTCAAAAGAGCATAGCATCACTAATGGA

***tm-1 VC532***  561 GGGTGCAGCATTTGCTGGAATGGTGATTGGAAGGCTTGAAAGTTCAAAAGAGCATAGCATCACTAATGGA

***Tm-1 VC554 2nd***  561 GGGTGCAGCATTTGCTGGAATGGTGATCGGAAGGCTTGAAACTTCAAAAGAGAATAGCATCACTACTGGA

***Tm-1 GCR237*** 631 AAGTTTACAGTTGGTGTAACTATGTTTGGGGTTACGACTCCTTGTGTTAATGCTGTCAAAGAAAGATTAG

***Tm-1 VC554 1st*** 631 AAGTTTACAGTTGGTGTAACTATGTTTGGGGTTACGACTCCTTGTGTTAATGCTGTCAAAGAAAGATTAG

***tm-1 GCR26***  631 AAGTTTACAGTTGGTGTAACTATGTTTGGGGTTACGACTCCTTGTGTTAATGCTGTCAAAGAAAGATTAG

***tm-1 Moneymaker*** 631 AAGTTTACAGTTGGTGTAACTATGTTTGGGGTTACGACTCCTTGTGTTAATGCTGTCAAAGAAAGATTAG

***tm-1 VC532***  631 AAGTTTACAGTTGGTGTAACTATGTTTGGGGTTACGACTCCTTGTGTTAATGCTGTCAAAGAAAGATTAG

***Tm-1 VC554 2nd***  631 AAGTTTACAGTTGGTGTAACTATGTTTGGGGTTACGACTCCTTGTGTTAATGCTGTCAAAGAAAGATTAG

***Tm-1 GCR237*** 701 TGAAAGAAGGATATGAGACTTTGGTGTTCCATGCCACGGGTGTCGGGGGCAGGGCCATGGAGGATCTTGT

***Tm-1 VC554 1st*** 701 TGAAAGAAGGATATGAGACTTTGGTGTTCCATGCCACGGGTGTCGGGGGCAGGGCCATGGAGGATCTTGT

***tm-1 GCR26***  701 TGAAAGAAGGATATGAGACTTTGGTGTTCCATGCCACGGGTGTCGGGGGCAGGGCCATGGAGGATCTTGT

***tm-1 Moneymaker*** 701 TGAAAGAAGGATATGAGACTTTGGTGTTCCATGCCACGGGTGTCGGGGGCAGGGCCATGGAGGATCTTGT

***tm-1 VC532***  701 TGAAAGAAGGATATGAGACTTTGGTGTTCCATGCCACGGGTGTCGGGGGCAGGGCCATGGAGGATCTTGT

***Tm-1 VC554 2nd***  701 TGAAAGAAGGATATGAGACTTTGGTTTTCCATGCCACGGGTGTCGGGGGCAGGGCCATGGAGGATCTTGT

***Tm-1 GCR237*** 771 TAGAGGAGGTTTTATACAGGGTGTGCTGGATATTACGACAACTGAGGTTGCAGATTACGTAGTTGGAGGA

***Tm-1 VC554 1st*** 771 TAGAGGAGGTTTTATACAGGGTGTGCTGGATATTACGACAACTGAGGTTGCAGATTACGTAGTTGGAGGA

***tm-1 GCR26***  771 TAGAGGAGGTTTTATACAGGGTGTGCTGGATATTACGACAACTGAGGTTGCAGATTACGTAGTTGGAGGA

***tm-1 Moneymaker*** 771 TAGAGGAGGTTTTATACAGGGTGTGCTGGATATTACGACAACTGAGGTTGCAGATTACGTAGTTGGAGGA

***tm-1 VC532***  771 TAGAGGAGGTTTTATACAGGGTGTGCTGGATATTACGACAACTGAGGTTGCAGATTACGTAGTTGGAGGA

***Tm-1 VC554 2nd***  771 TAGAGCAGGTTTTATACAGGGCGTGCTGGATATTACGACAACTGAGGTTGCAGATTACGTAGTTGGAGGA

***Tm-1 GCR237*** 841 GTAATGGCATGTGATAGTTCCCGATTTGATGCAATATTAGAGAAGAAAATTCCTTTGGTTCTGAGTGTGG

***Tm-1 VC554 1st*** 841 GTAATGGCATGTGATAGTTCCCGATTTGATGCAATATTAGAGAAGAAAATTCCTTTGGTTCTGAGTGTGG

***tm-1 GCR26***  841 GTAATGGCATGTGATAGTTCCCGATTTGATGCAATATTAGAGAAGAAAATTCCTTTGGTTCTGAGTGTGG

***tm-1 Moneymaker*** 841 GTAATGGCATGTGATAGTTCCCGATTTGATGCAATATTAGAGAAGAAAATTCCTTTGGTTCTGAGTGTGG

***tm-1 VC532***  841 GTAATGGCATGTGATAGTTCCCGATTTGATGCAATATTAGAGAAGAAAATTCCTTTGGTTCTGAGTGTGG

***Tm-1 VC554 2nd***  841 GTAATGGCATGTGATAGTTCCCGATTTGATGCAATATTAGAGAAGAAAATTCCTTTGGTTCTGAGTGTGG

***Tm-1 GCR237*** 911 GAGCACTGGATATGGTGAATTTTGGTCCTAAAACTACCATACCTCCTGAGTTTCAACAAAGAAAGATCCA

***Tm-1 VC554 1st*** 911 GAGCACTGGATATGGTGAATTTTGGTCCTAAAACTACCATACCTCCTGAGTTTCAACAAAGAAAGATCCA

***tm-1 GCR26***  911 GAGCACTGGATATGGTGAATTTTGGTCCTAAAACTACCATACCTCCTGAGTTTCAGCAAAGAAAGATTCA

***tm-1 Moneymaker*** 911 GAGCACTGGATATGGTGAATTTTGGTCCTAAAACTACCATACCTCCTGAGTTTCAGCAAAGAAAGATTCA

***tm-1 VC532***  911 GAGCACTGGATATGGTGAATTTTGGTCCTAAAACTACCATACCTCCTGAGTTTCAGCAAAGAAAGATTCA

***Tm-1 VC554 2nd***  911 GAGCACTGGATATGGTGAATTTTGGTCCTAAAACTACCATACCACCTGAGTTTCAGCAAAGAAAGATTCA

***Tm-1 GCR237*** 981 TGAACATAATGAGCAGGTTTCCCTAATGCGTACTACAGTAGGTGAAAATAAGAAATTTGCTGCATTTATA

***Tm-1 VC554 1st*** 981 TGAACATAATGAGCAGGTTTCCCTAATGCGTACTACAGTAGGTGAAAATAAGAAATTTGCTGCATTTATA

***tm-1 GCR26***  981 TCAACATAATGAGCAGGTTTCCCTAATGCATACTACAGTAGGTGAAAATAAGAAATTTGCTGCATTTATA

***tm-1 Moneymaker*** 981 TCAACATAATGAGCAGGTTTCCCTAATGCATACTACAGTAGGTGAAAATAAGAAATTTGCTGCATTTATA

***tm-1 VC532***  981 TCAACATAATGAGCAGGTTTCCCTAATGCGTACTACAGTAGGTGAAAATAAGAAATTTGCTGCATTTATA

***Tm-1 VC554 2nd***  981 TCAACATAATGAGCAGGTTTCCATAATGCGTACTACAGTAGGTGAAAATAAGAAATTTGCTGCATTTATA

***Tm-1 GCR237*** 1051 GCAGAAAAGTTGAACAAGGCATCATCAAGTGTATGTGTTTGCTTGCCAGAGAAAGGCGTGTCTGCATTGG

***Tm-1 VC554 1st*** 1051 GCAGAAAAGTTGAACAAGGCATCATCAAGTGTATGTGTTTGCTTGCCAGAGAAAGGCGTGTCTGCATTGG

***tm-1 GCR26***  1051 GCAGAAAAGTTGAACAAGGCATCATCAAGTGTATGTGTTTGCTTGCCAGAGAAAGGCGTGTCTGCATTGG

***tm-1 Moneymaker*** 1051 GCAGAAAAGTTGAACAAGGCATCATCAAGTGTATGTGTTTGCTTGCCAGAGAAAGGCGTGTCTGCATTGG

***tm-1 VC532***  1051 GCAGAAAAGTTGAACAAGGCATCATCAAGTGTATGTGTTTGCTTGCCAGAGAAAGGCGTGTCTGCATTGG

***Tm-1 VC554 2nd***  1051 GCTGAAAAGTTGAACAAGGCATCATCAAGTGTATGTGTTTGCTTGCCAGAGAAAGGTGTGTCTGCATTGG

***Tm-1 GCR237*** 1121 ATGCACCCGGGAAAGACTTTTATGATCCTGAGGCAACTAGTTGTCTTACACGTGAACTACAGATGCTTCT

***Tm-1 VC554 1st*** 1121 ATGCACCCGGGAAAGACTTTTATGATCCTGAGGCAACTAGTTGTCTTACACGTGAACTACAGATGCTTCT

***tm-1 GCR26***  1121 ATGCACCCGGGAAAGACTTTTATGATCCTGAGGCAACTAGTTGTCTTACACATGAACTACAGATGCTTCT

***tm-1 Moneymaker*** 1121 ATGCACCCGGGAAAGACTTTTATGATCCTGAGGCAACTAGTTGTCTTACACATGAACTACAGATGCTTCT

***tm-1 VC532***  1121 ATGCACCCGGGAAAGACTTTTATGATCCTGAGGCAACTAGTTGTCTTACACATGAACTACAGATGCTTCT

***Tm-1 VC554 2nd***  1121 ATGCACCGGGGAAAGAATTTTATGATCCTGAGGCAACTAGTTGTCTTACACATGAGCTACTGATGCTTCT

***Tm-1 GCR237*** 1191 TGAAAATAATGAACGTTGTCAGGTTAAGGTCCTCCCTTACCATATCAATGATGCGGAGTTTGCAAATGCT

***Tm-1 VC554 1st*** 1191 TGAAAATAATGAACGTTGTCAGGTTAAGGTCCTCCCTTACCATATCAATGATGCGGAGTTTGCAAATGCT

***tm-1 GCR26***  1191 TGAAAATAATGAACGTTGTCAGGTTAAGGTCTACCCTTACCATATCAATGATGTGGAGTTTGCAAATGCT

***tm-1 Moneymaker*** 1191 TGAAAATAATGAACGTTGTCAGGTTAAGGTCTACCCTTACCATATCAATGATGTGGAGTTTGCAAATGCT

***tm-1 VC532***  1191 TGAAAATAATGAACGTTGTCAGGTTAAGGTCTACCCTTACCATATCAATGATGTGGAGTTTGCAAATGCT

***Tm-1 VC554 2nd***  1191 TGAAAACAATGAACGTTGTCAGGTTAAGGTCTTCCCTTGCCATATCAATGATGCGGAGTTTGCAAATGCT

***Tm-1 GCR237*** 1261 TTAGTTGATTCATTCTTGGAAATCTCTCCGAAATCTAGACACGTAGAATGTCAGCCAGCTGAGTCCAAAT

***Tm-1 VC554 1st*** 1261 TTAGTTGATTCATTCTTGGAAATCTCTCCGAAATCTAGACACGTAGAATGTCAGCCAGCTGAGTCCAAAT

***tm-1 GCR26***  1261 TTAGTTGATTCATTTTTGGAAATGTCTCCGAAATCTGGACACGTAGAATGTCAGACAGCTGAGTCCAAAT

***tm-1 Moneymaker*** 1261 TTAGTTGATTCATTTTTGGAAATGTCTCCGAAATCTGGACACGTAGAATGTCAGACAGCTGAGTCCAAAT

***tm-1 VC532***  1261 TTAGTTGATTCATTTTTGGAAATGTCTCCGAAATCTGGACACGTAGAATGTCAGACAGCTGAGTCCAAAT

***Tm-1 VC554 2nd***  1261 TTAGTTGATTCATTCTTGGAAGTCTCTCCGAAATCTAGACACGTAGAATGTCAGCCAGCTGAGTCCAAAT

***Tm-1 GCR237*** 1331 CTATCCAAGACATTCAGAATGATAATGCTGTTCTAGAGAAATATCCCTCATGCAACGGGAAAAACTTTTC

***Tm-1 VC554 1st*** 1331 CTATCCAAGACATTCAGAATGATAATGCTGTTCTAGAGAAATATCCCTCATGCAACGGGAAAAACTTTTC

***tm-1 GCR26***  1331 CTATACAAGGCATTCAGAATGTTAATGCTGTTCTAGAGAAATATCCCTCATGCAACGGGAAAAACTTTTC

***tm-1 Moneymaker*** 1331 CTATACAAGGCATTCAGAATGTTAATGCTGTTCTAGAGAAATATCCCTCATGCAACGGGAAAAACTTTTC

***tm-1 VC532***  1331 CTATACAAGGCATTCAGAATGTTAATGCTGTTCTAGAGAAATATCCCTCATGCAACGGGAAAAACTTTTC

***Tm-1 VC554 2nd***  1331 GTATCCAAGACATTCAGAATGATAATGCTGTTCTAGAGAAATATCCCTCATGCAACGGGAAAAACTTTTC

***Tm-1 GCR237*** 1401 TCGCCTGAATGACTTTCCAAATGCAAAACCAGAAACTTTGCAGAAAAGAACTGTGATACTGCAGAAATTG

***Tm-1 VC554 1st*** 1401 TCGCCTGAATGACTTTCCAAATGCAAAACCAGAAACTTTGCAGAAAAGAACTGTGATACTGCAGAAATTG

***tm-1 GCR26***  1401 TCGCCTGAATGACTTTCCAAATGCAAAACCAGAAACTTTGCAGAAAAGAATTGTGATACTGCAGAAATTG

***tm-1 Moneymaker*** 1401 TCGCCTGAATGACTTTCCAAATGCAAAACCAGAAACTTTGCAGAAAAGAATTGTGATACTGCAGAAATTG

***tm-1 VC532***  1401 TCGCCTGAATGACTTTCCAAATGCAAAACCAGAAACTTTGCAGAAAAGAATTGTGATACTGCAGAAATTG

***Tm-1 VC554 2nd***  1401 TCGCCTGAATGACTTTCCAAATGCAAAACCAGAAACTTTGCAGAAAAGAACTGTGATACTGCAGAAATTG

***Tm-1 GCR237*** 1471 AAAGATCAAATAAGTAAGGGCAAGCCTATTATTGGGGCTGGTGCTGGTACAGGTATTTCTGCTAAGTTTG

***Tm-1 VC554 1st*** 1471 AAAGATCAAATAAGTAAGGGCAAGCCTATTATTGGGGCTGGTGCTGGTACAGGTATTTCTGCTAAGTTTG

***tm-1 GCR26***  1471 AAAGATCAAATAAGTAAGGGCAAGCCTATTATTGGGGCTGGTGCTGGTACAGGTATTTCTGCTAAGTTTG

***tm-1 Moneymaker*** 1471 AAAGATCAAATAAGTAAGGGCAAGCCTATTATTGGGGCTGGTGCTGGTACAGGTATTTCTGCTAAGTTTG

***tm-1 VC532***  1471 AAAGATCAAATAAGTAAGGGCAAGCCTATTATTGGGGCTGGTGCTGGTACAGGTATTTCTGCTAAGTTTG

***Tm-1 VC554 2nd***  1471 AAAGATCAAATAAGTAAGGGCAAGCCTATTATTGGGGCTGGAGCTGGTACAGGTATTTCTGCTAAGTTTG

***Tm-1 GCR237***  1541 AGGAAGCTGGTGGTGTAGATTTGATTGTCTTGTACAACTCAGGGCGCTTTAGGATGGCAGGAAGGGGATC

***Tm-1 VC554 1st*** 1541 AGGAAGCTGGTGGTGTAGATTTGATTGTCTTGTACAACTCAGGGCGCTTTAGGATGGCAGGAAGGGGATC

***tm-1 GCR26***  1541 AGGAAGCTGGTGGTGTAGATTTGATTGTCTTGTACAACTCAGGGCGCTTTAGGATGGCAGGAAGGGGATC

***tm-1 Moneymaker*** 1541 AGGAAGCTGGTGGTGTAGATTTGATTGTCTTGTACAACTCAGGGCGCTTTAGGATGGCAGGAAGGGGATC

***tm-1 VC532***  1541 AGGAAGCTGGTGGTGTAGATTTGATTGTCTTGTACAACTCAGGGCGCTTTAGGATGGCAGGAAGGGGATC

***Tm-1 VC554 2nd***  1541 AGGAAGCTGGTGGTGTGGATTTGATTGTCTTGTACAACTCAGGGCGCTTTAGGATGGCAGGAAGGGGATC

***Tm-1 GCR237*** 1611 CTTAGCTGGTCTACTGCCCTTTGCTGATGCAAATGCCATTGTACTTGAGATGGCCAACGAAGTATTGCCT

***Tm-1 VC554 1st*** 1611 CTTAGCTGGTCTACTGCCCTTTGCTGATGCAAATGCCATTGTACTTGAGATGGCCAACGAAGTATTGCCT

***tm-1 GCR26***  1611 CTTAGCTGGTCTATTGCCCTTTGCTGATGCAAATGCCATTGTACTTGAGATGGCCAACGAAGTATTGCCT

***tm-1 Moneymaker*** 1611 CTTAGCTGGTCTATTGCCCTTTGCTGATGCAAATGCCATTGTACTTGAGATGGCCAACGAAGTATTGCCT

***tm-1 VC532***  1611 CTTAGCTGGTCTATTGCCCTTTGCTGATGCAAATGCCATTGTACTTGAGATGGCCAACGAAGTATTGCCT

***Tm-1 VC554 2nd***  1611 CTTAGCTGGTCTATTGCCCTTTGCTGATGCAAATGCCATTGTACTTGAGATGGCCAACGAAGTATTGCCG

***Tm-1 GCR237*** 1681 GTGGTTAAGGAAGTGGCAGTTCTGGCTGGAGTTTGTGCTACTGATCCTTTCCGCAGGATGGACAACTTCC

***Tm-1 VC554 1st*** 1681 GTGGTTAAGGAAGTGGCAGTTCTGGCTGGAGTTTGTGCTACTGATCCTTTCCGCAGGATGGACAACTTCC

***tm-1 GCR26***  1681 GTGGTTAAGGAAGTGGCAGTTCTGGCTGGAGTTTGTGCTACTGATCCTTTCCGCAGGATGGACAACTTCC

***tm-1 Moneymaker*** 1681 GTGGTTAAGGAAGTGGCAGTTCTGGCTGGAGTTTGTGCTACTGATCCTTTCCGCAGGATGGACAACTTCC

***tm-1 VC532***  1681 GTGGTTAAGGAAGTGGCAGTTCTGGCTGGAGTTTGTGCTACTGATCCTTTCCGCAGGATGGACAACTTCC

***Tm-1 VC554 2nd***  1681 GTGGTTAAGGAAGTGGCAGTTCTGGCTGGAGTTTGTGCAACTGATCCTTTCCGCAGGATGGACAACTTCC

***Tm-1 GCR237*** 1751 TGAAGCAGTTGGAATCCGTTGGATTCTGTGGGGTGCAAAACTTTCCAACTGTTGGTCTGTTTGACGGTAA

***Tm-1 VC554 1st*** 1751 TGAAGCAGTTGGAATCCGTTGGATTCTGTGGGGTGCAAAACTTTCCAACTGTTGGTCTGTTTGACGGTAA

***tm-1 GCR26***  1751 TGAAGCAGTTGGAATCTGTTGGATTCTGTGGGGTGCAAAACTTTCCAACTGTTGGTCTGTTTGACGGTAA

***tm-1 Moneymaker*** 1751 TGAAGCAGTTGGAATCTGTTGGATTCTGTGGGGTGCAAAACTTTCCAACTGTTGGTCTGTTTGACGGTAA

***tm-1 VC532***  1751 TGAAGCAGTTGGAATCTGTTGGATTCTGTGGGGTGCAAAACTTTCCAACTGTTGGTCTGTTTGACGGTAA

***Tm-1 VC554 2nd***  1751 TGAAGCAGTTGGAATCCGTTGGATTCTGTGGGGTGCAAAACTTTCCAACTGTTGGTCTGTTTGACGGTAA

***Tm-1 GCR237*** 1821 CTTCAGACAAAATTTGGAAGAGACTGGAATGGGTTATGGCTTGGAGGTTGAGATGATTGCAGCAGCTCAC

***Tm-1 VC554 1st*** 1821 CTTCAGACAAAATTTGGAAGAGACTGGAATGGGTTATGGCTTGGAGGTTGAGATGATTGCAGCAGCTCAC

***tm-1 GCR26***  1821 CTTCAGACAAAATTTGGAAGAGACTGGAATGGGTTATGGCTTGGAGGTTGAGATGATTGCAACAGCTCAT

***tm-1 Moneymaker*** 1821 CTTCAGACAAAATTTGGAAGAGACTGGAATGGGTTATGGCTTGGAGGTTGAGATGATTGCAACAGCTCAT

***tm-1 VC532***  1821 CTTCAGACAAAATTTGGAAGAGACTGGAATGGGTTATGGCTTGGAGGTTGAGATGATTGCAACAGCTCAT

***Tm-1 VC554 2nd***  1821 CTTCAGACAAAATTTGGAAGAGACTGGAATGGGTTATGGCTTGGAGGTTGAGATGATTGCAACAGCTCAC

***Tm-1 GCR237***  1891 AGGATGGGCCTTTTGACAACCCCATATGCTTTCTGCCCAGATGAAGCAGTTGCTATGGCAGAAGCTGGTG

***Tm-1 VC554 1st*** 1891 AGGATGGGCCTTTTGACAACCCCATATGCTTTCTGCCCAGATGAAGCAGTTGCTATGGCAGAAGCTGGTG

***tm-1 GCR26***  1891 AGGATGGGCCTTTTGACAACCCCATATGCTTTCTGCCCAGATGAAGCAGTTGCTATGGCAGAAGCTGGTG

***tm-1 Moneymaker*** 1891 AGGATGGGCCTTTTGACAACCCCATATGCTTTCTGCCCAGATGAAGCAGTTGCTATGGCAGAAGCTGGTG

***tm-1 VC532***  1891 AGGATGGGCCTTTTGACAACCCCATATGCTTTCTGCCCAGATGAAGCAGTTGCTATGGCAGAAGCTGGTG

***Tm-1 VC554 2nd***  1891 AGGATGGGCCTTTTGACAACCCCATATGCTTTCTGCCCAGATGAAGCAGTTGCTATGGCAGAAGCTGGTG

***Tm-1 GCR237*** 1961 CCGACATCATAGTTGCTCATATGGGGCTTACAACATCTGGTTCAATTGGTGCAAAAACAGCCGTCTCATT

***Tm-1 VC554 1st*** 1961 CCGACATCATAGTTGCTCATATGGGGCTTACAACATCTGGTTCAATTGGTGCAAAAACAGCCGTCTCATT

***tm-1 GCR26***  1961 CCGACATCATAGTTGCTCATATGGGGCTTACAACATCTGGTTCAATTGGTGCAAAAACAGCTGTATCATT

***tm-1 Moneymaker*** 1961 CCGACATCATAGTTGCTCATATGGGGCTTACAACATCTGGTTCAATTGGTGCAAAAACAGCTGTATCATT

***tm-1 VC532***  1961 CCGACATCATAGTTGCTCATATGGGGCTTACAACATCTGGTTCAATTGGTGCAAAAACAGCTGTATCATT

***Tm-1 VC554 2nd***  1961 CCGACATCATAGTTGCTCATATGGGGCTTACAACATCTGGTTCAATTGGTGCAAAAACAGCTGTCTCATT

***Tm-1 GCR237*** 2031 GGAGGAAAGTGTAACTTGCGTTCAAGCTATTGCAGATGCTACTCATAGGATATATCCTGATGCAATTGTG

***Tm-1 VC554 1st*** 2031 GGAGGAAAGTGTAACTTGCGTTCAAGCTATTGCAGATGCTACTCATAGGATATATCCTGATGCAATTGTG

***tm-1 GCR26***  2031 GGAGGAAAGTGTAACTTGCGTCCAAGCTATTGCAGATGCTACTCATAGGATAAATCCTGATGCAATTGTG

***tm-1 Moneymaker*** 2031 GGAGGAAAGTGTAACTTGCGTCCAAGCTATTGCAGATGCTACTCATAGGATAAATCCTGATGCAATTGTG

***tm-1 VC532***  2031 GGAGGAAAGTGTAACTTGCGTCCAAGCTATTGCAGATGCTACTCATAGGATAAATCCTGATGCAATTGTG

***Tm-1 VC554 2nd***  2031 GGAGGAAAGTGTAACTTGCGTCCAAGCTATTGCAGATGCTACTCATAGGATAAATCCTGATGCAATTGTG

***Tm-1 GCR237*** 2101 CTCTGCCATGGAGGCCCTATATCTTCCCCTGAAGAAGCAGCATATGTACTGAAGAGAACCACAGGAGTTC

***Tm-1 VC554 1st*** 2101 CTCTGCCATGGAGGCCCTATATCTTCCCCTGAAGAAGCAGCATATGTACTGAAGAGAACCACAGGAGTTC

***tm-1 GCR26***  2101 CTCTGCCATGGAGGCCCTATATCTTCCCCTGAAGAAGCAGCATATGTACTGAAGAGAACCACAGGAGTTC

***tm-1 Moneymaker*** 2101 CTCTGCCATGGAGGCCCTATATCTTCCCCTGAAGAAGCAGCATATGTACTGAAGAGAACCACAGGAGTTC

***tm-1 VC532***  2101 CTCTGCCATGGAGGCCCTATATCTTCCCCTGAAGAAGCAGCATATGTACTGAAGAGAACCACAGGAGTTC

***Tm-1 VC554 2nd***  2101 CTCTGCCATGGAGGCCCTATATCTTCCCCTGAAGAAGCAGCATATGTACTGAAGAGAACCACAGGAGTTC

***Tm-1 GCR237*** 2171 ATGGATTTTATGGCGCTTCAAGCATGGAAAGACTACCAGTTGAGCAAGCTATAACTGCAACTGTCCAGCA

***Tm-1 VC554 1st*** 2171 ATGGATTTTATGGCGCTTCAAGCATGGAAAGACTACCAGTTGAGCAAGCTATAACTGCAACTGTCCAGCA

***tm-1 GCR26***  2171 ATGGATTTTATGGCGCTTCAAGCATGGAAAGACTACCAGTTGAGCAAGCTATAACTGCAACTGTCCAACA

***tm-1 Moneymaker*** 2171 ATGGATTTTATGGCGCTTCAAGCATGGAAAGACTACCAGTTGAGCAAGCTATAACTGCAACTGTCCAACA

***tm-1 VC532***  2171 ATGGATTTTATGGCGCTTCAAGCATGGAAAGACTACCAGTTGAGCAAGCTATAACTGCAACTGTCCAACA

***Tm-1 VC554 2nd***  2171 ATGGATTTTATGGCGCTTCAAGCATGGAAAGACTACCAGTTGAGCAAGCTATAACTGCAACTGTCCAGCA

***Tm-1 GCR237*** 2241 GTACAAGTCTATTTCTATGGAGTGA

***Tm-1 VC554 1st*** 2241 GTACAAGTCTATTTCTATGGAGTGA

***tm-1 GCR26***  2241 GTACAAGTCTATATCTATGGAGTGA

***tm-1 Moneymaker*** 2241 GTACAAGTCTATATCTATGGAGTGA

***tm-1 VC532***  2241 GTACAAGTCTATATCTATGGAGTGA

***Tm-1 VC554 2nd***  2241 GTACAAGTCTATTTCTATGGAGTGA
