# Supplementary material for: Contribution of the tobamovirus resistance gene Tm-1 to control of tomato brown rugose fruit virus (ToBRFV) resistance in tomato
Source: PLoS Genet. 2025 May 23;21(5):e1011725. doi: 10.1371/journal.pgen.1011725 (PMC12140429; doi:10.1371/journal.pgen.1011725)
Supplement: S2 Fig — (DOCX) [file pgen.1011725.s002.docx]

**S2 Fig. *Tm-1* amino acid sequence alignment.**

***Tm-1 GCR237***  1 MATAQSNSPRVFCIGTADTKFDELRFLSEHVRSSLNSFSNKSSFKVGVTVVDVSTSWKETNSCADFDFVP

***Tm-1 VC554 1st***  1 MATAQSNSPRVFCIGTADTKFDELRFLSEHVRSSLNSFSNKSSFKVGVTVVDVSTSWKETNSCADFDFVP

***tm-1 GCR26***  1 MATAQSNSPRVFCIGTADTKFDELRFLSEHVRSSLNSFSNKSSFKVGVTVVDVSTSRKETNSCADFDFVP

***tm-1 Moneymaker***  1 MATAQSNSPRVFCIGTADTKFDELRFLSEHVRSSLNSFSNKSSFKVGVTVVDVSTSRKETNSCADFDFVP

***tm-1 VC532***  1 MATAQSNSPRVFCIGTADTKFDELRFLSEHVRSSLNSFSNKSSFKVGVTVVDVSTSRKETNSCADFDFVP

***Tm-1 VC554 2nd***  1 MASAQSNSPRVFCIGTADTKFDELRFLSQYVRSSLNSFSNKSSFKVGVTVVDVSTSLKETNGCADFDFVP

***Tm-1 GCR237***  71 SKDVLSCHTLGEETMGTFADIRGLAIAIMSKALETFLSIANDEQNLAGVIGLGGSGGTSLLSSAFRSLPI

***Tm-1 VC554 1st***  71 SKDVLSCHTLGEETMGTFADIRGLAIAIMSKALETFLSIANDEQNLAGVIGLGGSGGTSLLSSAFRSLPI

***tm-1 GCR26***  71 SKDVLSCYARGEGTVGRFPDIRGQAIAIMNKALETFLSKANGEQNLAGVIGLGGSGGTSLLSSAFRSLPI

***tm-1 Moneymaker***  71 SKDVLSCYARGEGTVGRFPDIRGQAIAIMNKALETFLSKANGEQNLAGVIGLGGSGGTSLLSSAFRSLPI

***tm-1 VC532***  71 SKDVLSCYARGEGTVGRFPDIRGQAIAIMNKALETFLSKANGEQNLAGVIGLGGSGGTSLLSSAFRSLPI

***Tm-1 VC554 2nd***  71 RKDVLSCYAQGGESVVQLPDDRGQAIAIMNKAFQTFLSKANGEQNLAGVIGLGGSGGTSLLSSAFRSLPI

***Tm-1 GCR237***  141 GIPKVIISTVASGQTESYIGTSDLVLFPSVVDICGINNVSKVVLSNAGAAFAGMVIGRLESSKEHSITNG

***Tm-1 VC554 1st***  141 GIPKVIISTVASGQTESYIGTSDLVLFPSVVDICGINNVSKVVLSNAGAAFAGMVIGRLESSKEHSITNG

***tm-1 GCR26***  141 GIPKVIISTVASGQTESYIGTSDLVLFPSVVDICGINNVSKVVLSNAGAAFAGMVIGRLESSKEHSITNG

***tm-1 Moneymaker***  141 GIPKVIISTVASGQTESYIGTSDLVLFPSVVDICGINNVSKVVLSNAGAAFAGMVIGRLESSKEHSITNG

***tm-1 VC532***  141 GIPKVIISTVASGQTESYIGTSDLVLFPSVVDICGINNVSKVVLSNAGAAFAGMVIGRLESSKEHSITNG

***Tm-1 VC554 2nd***  141 GIPKVIISTVASGQTESYIGTSDLVLFPSVVDICGINNVSKVILSNAGAAFAGMVIGRLETSKENSITTG

***Tm-1 GCR237***  211 KFTVGVTMFGVTTPCVNAVKERLVKEGYETLVFHATGVGGRAMEDLVRGGFIQGVLDITTTEVADYVVGG

***Tm-1 VC554 1st***  211 KFTVGVTMFGVTTPCVNAVKERLVKEGYETLVFHATGVGGRAMEDLVRGGFIQGVLDITTTEVADYVVGG

***tm-1 GCR26***  211 KFTVGVTMFGVTTPCVNAVKERLVKEGYETLVFHATGVGGRAMEDLVRGGFIQGVLDITTTEVADYVVGG

***tm-1 Moneymaker***  211 KFTVGVTMFGVTTPCVNAVKERLVKEGYETLVFHATGVGGRAMEDLVRGGFIQGVLDITTTEVADYVVGG

***tm-1 VC532***  211 KFTVGVTMFGVTTPCVNAVKERLVKEGYETLVFHATGVGGRAMEDLVRGGFIQGVLDITTTEVADYVVGG

***Tm-1 VC554 2nd***  211 KFTVGVTMFGVTTPCVNAVKERLVKEGYETLVFHATGVGGRAMEDLVRAGFIQGVLDITTTEVADYVVGG

***Tm-1 GCR237***  281 VMACDSSRFDAILEKKIPLVLSVGALDMVNFGPKTTIPPEFQQRKIHEHNEQVSLMRTTVGENKKFAAFI

***Tm-1 VC554 1st***  281 VMACDSSRFDAILEKKIPLVLSVGALDMVNFGPKTTIPPEFQQRKIHEHNEQVSLMRTTVGENKKFAAFI

***tm-1 GCR26***  281 VMACDSSRFDAILEKKIPLVLSVGALDMVNFGPKTTIPPEFQQRKIHQHNEQVSLMHTTVGENKKFAAFI

***tm-1 Moneymaker***  281 VMACDSSRFDAILEKKIPLVLSVGALDMVNFGPKTTIPPEFQQRKIHQHNEQVSLMHTTVGENKKFAAFI

***tm-1 VC532***  281 VMACDSSRFDAILEKKIPLVLSVGALDMVNFGPKTTIPPEFQQRKIHQHNEQVSLMRTTVGENKKFAAFI

***Tm-1 VC554 2nd***  281 VMACDSSRFDAILEKKIPLVLSVGALDMVNFGPKTTIPPEFQQRKIHQHNEQVSIMRTTVGENKKFAAFI

***Tm-1 GCR237***  351 AEKLNKASSSVCVCLPEKGVSALDAPGKDFYDPEATSCLTRELQMLLENNERCQVKVLPYHINDAEFANA

***Tm-1 VC554 1st***  351 AEKLNKASSSVCVCLPEKGVSALDAPGKDFYDPEATSCLTRELQMLLENNERCQVKVLPYHINDAEFANA

***tm-1 GCR26***  351 AEKLNKASSSVCVCLPEKGVSALDAPGKDFYDPEATSCLTHELQMLLENNERCQVKVYPYHINDVEFANA

***tm-1 Moneymaker***  351 AEKLNKASSSVCVCLPEKGVSALDAPGKDFYDPEATSCLTHELQMLLENNERCQVKVYPYHINDVEFANA

***tm-1 VC532***  351 AEKLNKASSSVCVCLPEKGVSALDAPGKDFYDPEATSCLTHELQMLLENNERCQVKVYPYHINDVEFANA

***Tm-1 VC554 2nd***  351 AEKLNKASSSVCVCLPEKGVSALDAPGKEFYDPEATSCLTHELLMLLENNERCQVKVFPCHINDAEFANA

***Tm-1 GCR237***  421 LVDSFLEISPKSRHVECQPAESKSIQDIQNDNAVLEKYPSCNGKNFSRLNDFPNAKPETLQKRTVILQKL

***Tm-1 VC554 1st***  421 LVDSFLEISPKSRHVECQPAESKSIQDIQNDNAVLEKYPSCNGKNFSRLNDFPNAKPETLQKRTVILQKL

***tm-1 GCR26***  421 LVDSFLEMSPKSGHVECQTAESKSIQGIQNVNAVLEKYPSCNGKNFSRLNDFPNAKPETLQKRIVILQKL

***tm-1 Moneymaker***  421 LVDSFLEMSPKSGHVECQTAESKSIQGIQNVNAVLEKYPSCNGKNFSRLNDFPNAKPETLQKRIVILQKL

***tm-1 VC532***  421 LVDSFLEMSPKSGHVECQTAESKSIQGIQNVNAVLEKYPSCNGKNFSRLNDFPNAKPETLQKRIVILQKL

***Tm-1 VC554 2nd***  421 LVDSFLEVSPKSRHVECQPAESKCIQDIQNDNAVLEKYPSCNGKNFSRLNDFPNAKPETLQKRTVILQKL

***Tm-1 GCR237***  491 KDQISKGKPIIGAGAGTGISAKFEEAGGVDLIVLYNSGRFRMAGRGSLAGLLPFADANAIVLEMANEVLP

***Tm-1 VC554 1st***  491 KDQISKGKPIIGAGAGTGISAKFEEAGGVDLIVLYNSGRFRMAGRGSLAGLLPFADANAIVLEMANEVLP

***tm-1 GCR26***  491 KDQISKGKPIIGAGAGTGISAKFEEAGGVDLIVLYNSGRFRMAGRGSLAGLLPFADANAIVLEMANEVLP

***tm-1 Moneymaker***  491 KDQISKGKPIIGAGAGTGISAKFEEAGGVDLIVLYNSGRFRMAGRGSLAGLLPFADANAIVLEMANEVLP

***tm-1 VC532***  491 KDQISKGKPIIGAGAGTGISAKFEEAGGVDLIVLYNSGRFRMAGRGSLAGLLPFADANAIVLEMANEVLP

***Tm-1 VC554 2nd***  491 KDQISKGKPIIGAGAGTGISAKFEEAGGVDLIVLYNSGRFRMAGRGSLAGLLPFADANAIVLEMANEVLP

***Tm-1 GCR237***  561 VVKEVAVLAGVCATDPFRRMDNFLKQLESVGFCGVQNFPTVGLFDGNFRQNLEETGMGYGLEVEMIAAAH

***Tm-1 VC554 1st***  561 VVKEVAVLAGVCATDPFRRMDNFLKQLESVGFCGVQNFPTVGLFDGNFRQNLEETGMGYGLEVEMIAAAH

***tm-1 GCR26***  561 VVKEVAVLAGVCATDPFRRMDNFLKQLESVGFCGVQNFPTVGLFDGNFRQNLEETGMGYGLEVEMIATAH

***tm-1 Moneymaker***  561 VVKEVAVLAGVCATDPFRRMDNFLKQLESVGFCGVQNFPTVGLFDGNFRQNLEETGMGYGLEVEMIATAH

***tm-1 VC532***  561 VVKEVAVLAGVCATDPFRRMDNFLKQLESVGFCGVQNFPTVGLFDGNFRQNLEETGMGYGLEVEMIATAH

***Tm-1 VC554 2nd***  561 VVKEVAVLAGVCATDPFRRMDNFLKQLESVGFCGVQNFPTVGLFDGNFRQNLEETGMGYGLEVEMIATAH

***Tm-1 GCR237***  631 RMGLLTTPYAFCPDEAVAMAEAGADIIVAHMGLTTSGSIGAKTAVSLEESVTCVQAIADATHRIYPDAIV

***Tm-1 VC554 1st***  631 RMGLLTTPYAFCPDEAVAMAEAGADIIVAHMGLTTSGSIGAKTAVSLEESVTCVQAIADATHRIYPDAIV

***tm-1 GCR26***  631 RMGLLTTPYAFCPDEAVAMAEAGADIIVAHMGLTTSGSIGAKTAVSLEESVTCVQAIADATHRINPDAIV

***tm-1 Moneymaker***  631 RMGLLTTPYAFCPDEAVAMAEAGADIIVAHMGLTTSGSIGAKTAVSLEESVTCVQAIADATHRINPDAIV

***tm-1 VC532***  631 RMGLLTTPYAFCPDEAVAMAEAGADIIVAHMGLTTSGSIGAKTAVSLEESVTCVQAIADATHRINPDAIV

***Tm-1 VC554 2nd***  631 RMGLLTTPYAFCPDEAVAMAEAGADIIVAHMGLTTSGSIGAKTAVSLEESVTCVQAIADATHRINPDAIV

***Tm-1 GCR237***  701 LCHGGPISSPEEAAYVLKRTTGVHGFYGASSMERLPVEQAITATVQQYKSISME-

***Tm-1 VC554 1st***  701 LCHGGPISSPEEAAYVLKRTTGVHGFYGASSMERLPVEQAITATVQQYKSISME-

***tm-1 GCR26***  701 LCHGGPISSPEEAAYVLKRTTGVHGFYGASSMERLPVEQAITATVQQYKSISME-

***tm-1 Moneymaker***  701 LCHGGPISSPEEAAYVLKRTTGVHGFYGASSMERLPVEQAITATVQQYKSISME-

***tm-1 VC532***  701 LCHGGPISSPEEAAYVLKRTTGVHGFYGASSMERLPVEQAITATVQQYKSISME-

***Tm-1 VC554 2nd***  701 LCHGGPISSPEEAAYVLKRTTGVHGFYGASSMERLPVEQAITATVQQYKSISME-
