# Supplementary material for: Contribution of the tobamovirus resistance gene Tm-1 to control of tomato brown rugose fruit virus (ToBRFV) resistance in tomato
Source: PLoS Genet. 2025 May 23;21(5):e1011725. doi: 10.1371/journal.pgen.1011725 (PMC12140429; doi:10.1371/journal.pgen.1011725)
Supplement: S1 Table — (DOCX) [file pgen.1011725.s003.docx]

**S3 Table. Disease Severity Index (DSI) metrics show** **the** **number of plants in each DSI in the experiment analyzing the Association of *Tm-1* with Resistance phenotype in plants carrying the tolerance locus.**

|  | **Genotype** | **No. of plants showing DSI of** | | | | | | |
| --- | --- | --- | --- | --- | --- | --- | --- | --- |
|  |  | **0** | **0.5** | **1** | **1.5** | **2** | **2.5** | **3** |
| **Control lines** | Moneymaker |  |  |  |  |  |  | 4 |
|  | VC532 | 4 |  |  |  |  |  |  |
|  | VC554 | 4 |  |  |  |  |  |  |
| **F_3_ plants** | *11^VC532^/11^VC532^ tm-1/tm-1* | 16 |  |  |  |  |  |  |
|  | *11^VC532^/11^VC532^ Tm-1/tm-1* | 29 |  |  |  |  |  |  |
|  | *11^VC532^/11^VC532^ Tm-1/Tm-1* | 15 |  |  |  |  |  |  |
