# Supplementary material for: Contribution of the tobamovirus resistance gene Tm-1 to control of tomato brown rugose fruit virus (ToBRFV) resistance in tomato
Source: PLoS Genet. 2025 May 23;21(5):e1011725. doi: 10.1371/journal.pgen.1011725 (PMC12140429; doi:10.1371/journal.pgen.1011725)
Supplement: S2 Table — (DOCX) [file pgen.1011725.s004.docx]

**S4 Table. *Tm-1* transcription fold across VC532-*Tm-1*-OE T_0_ transgenic plants.**

| **Line** | **Description** | **Type** | ***Tm-1* Transcription Fold** |
| --- | --- | --- | --- |
| VC532 | *11^VC532^/11^VC532^,tm-1/tm-1* | Not Transgenic control | 1 |
| VC532-*Tm-1*-OE-1 | *Tm-1* Overexpression in VC532 | T_0_ Transgenic | 4 |
| VC532-*Tm-1*-OE-2 | *Tm-1* Overexpression in VC532 | T_0_ Transgenic | 200 |
| VC532-*Tm-1*-OE-3 | *Tm-1* Overexpression in VC532 | T_0_ Transgenic | 400 |
| VC532-*Tm-1*-OE-4 | *Tm-1* Overexpression in VC532 | T_0_ Transgenic | 180 |
| VC532-*Tm-1*-OE-5 | *Tm-1* Overexpression in VC532 | T_0_ Transgenic | 220 |
| VC532-*Tm-1*-OE-6 | *Tm-1* Overexpression in VC532 | T_0_ Transgenic | 10 |
| VC532- *Tm-1*-OE-7 | *Tm-1* Overexpression in VC532 | T_0_ Transgenic | 600 |
| VC532- *Tm-1*-OE-8 | *Tm-1* Overexpression in VC532 | T_0_ Transgenic | 90 |
| VC532- *Tm-1*-OE-9 | *Tm-1* Overexpression in VC532 | T_0_ Transgenic | 3 |
| VC532- *Tm-1*-OE-10 | *Tm-1* Overexpression in VC532 | T_0_ Transgenic | 1000 |
| VC532- *Tm-1*-OE-11 | *Tm-1* Overexpression in VC532 | T_0_ Transgenic | 400 |
