# Supplementary material for: Contribution of the tobamovirus resistance gene Tm-1 to control of tomato brown rugose fruit virus (ToBRFV) resistance in tomato
Source: PLoS Genet. 2025 May 23;21(5):e1011725. doi: 10.1371/journal.pgen.1011725 (PMC12140429; doi:10.1371/journal.pgen.1011725)
Supplement: S4 Table — (DOCX) [file pgen.1011725.s006.docx]

| **Line** | **Description** | **Type** | ***Tm-1* Transcription Fold** |
| --- | --- | --- | --- |
| Moneymaker | *11^MM^/11^MM^,tm-1/tm-1* | Not Transgenic control | 1 |
| Moneymaker-*Tm-1*-OE-1 | *Tm-1* Overexpression in Moneymaker | T_0_ Transgenic | 18 |
| Moneymaker- *Tm-1*-OE-2 | *Tm-1* Overexpression in Moneymaker | T_0_ Transgenic | 1 |
| Moneymaker-*Tm-1*-OE-3 | *Tm-1* Overexpression in Moneymaker | T_0_ Transgenic | 0.7 |
| Moneymaker-*Tm-1*-OE-4 | *Tm-1* Overexpression in Moneymaker | T_0_ Transgenic | 3 |
| Moneymaker-*Tm-1*-OE-5 | *Tm-1* Overexpression in Moneymaker | T_0_ Transgenic | 1.5 |
| Moneymaker-*Tm-1*-OE-6 | *Tm-1* Overexpression in Moneymaker | T_0_ Transgenic | 0.5 |
| Moneymaker-*Tm-1*-OE-7 | *Tm-1* Overexpression in Moneymaker | T_0_ Transgenic | 0.8 |
| Moneymaker-*Tm-1*-OE-8 | *Tm-1* Overexpression in Moneymaker | T_0_ Transgenic | 2.7 |
| Moneymaker-*Tm-1*-OE-9 | *Tm-1* Overexpression in Moneymaker | T_0_ Transgenic | 0.15 |
| Moneymaker-*Tm-1*-OE-10 | *Tm-1* Overexpression in Moneymaker | T_0_ Transgenic | 1.5 |
| Moneymaker-*Tm-1*-OE-11 | *Tm-1* Overexpression in Moneymaker | T_0_ Transgenic | 0.4 |
| Moneymaker-*Tm-1*-OE-12 | *Tm-1* Overexpression in Moneymaker | T_0_ Transgenic | 1.8 |
| Moneymaker-*Tm-1*-OE-13 | *Tm-1* Overexpression in Moneymaker | T_0_ Transgenic | 2.5 |
| Moneymaker-*Tm-1*-OE-14 | *Tm-1* Overexpression in Moneymaker | T_0_ Transgenic | 1.7 |
| Moneymaker-*Tm-1*-OE-15 | *Tm-1* Overexpression in Moneymaker | T_0_ Transgenic | 0.8 |
| Moneymaker-*Tm-1*-OE-16 | *Tm-1* Overexpression in Moneymaker | T_0_ Transgenic | 1.3 |
| Moneymaker-*Tm-1*-OE-17 | *Tm-1* Overexpression in Moneymaker | T_0_ Transgenic | 1.6 |

**S6 Table. *Tm-1* transcription fold across Moneymaker-*Tm-1*-OE T_0_ transgenic plants.**
