# Supplementary material for: Contribution of the tobamovirus resistance gene Tm-1 to control of tomato brown rugose fruit virus (ToBRFV) resistance in tomato
Source: PLoS Genet. 2025 May 23;21(5):e1011725. doi: 10.1371/journal.pgen.1011725 (PMC12140429; doi:10.1371/journal.pgen.1011725)
Supplement: S7 Table — (DOCX) [file pgen.1011725.s009.docx]

**S9 Table. Disease Severity Index (DSI) metrics show** **the** **number of plants in each DSI at 46 DPI in the experiment analyzing overexpression of *Tm-1* in the susceptible genotype Moneymaker.**

|  | **Line** | **Description** | **Type** | **No. of plants showing DSI at 45 DPI** | | | | | | |
| --- | --- | --- | --- | --- | --- | --- | --- | --- | --- | --- |
| **A.** | **Control genotypes** |  |  | **0** | **0.5** | **1** | **1.5** | **2** | **2.5** | **3** |
|  | Moneymaker | *11^MM^/11^MM^,tm-1/tm-1* | Non-transgenic control |  |  |  | 1 | 5 | 4 | 5 |
|  | LA2825 | *11^LA2825^/11^LA2825^,Tm-1/Tm-1* | Non-transgenic control |  |  |  | 2 | 3 | 3 | 7 |
| **B.** | **Original T_1_ parental lines** |  |  |  |  |  |  |  |  |  |
|  | TM-184 | *11^MM^/11^MM^,Tm-1^OE^/Tm-1^OE^* | T_2_ transgenic | 7 | 4 | 2 | 1 | 1 |  |  |
|  |  | *11^MM^/11^MM^,tm-1/tm-1* | T_2_ azygous control |  |  |  |  | 1 | 5 | 9 |
|  | TM-185 | *11^MM^/11^MM^,Tm-1^OE^/Tm-1^OE^* | T_2_ transgenic | 4 | 2 | 2 | 2 | 2 | 1 | 2 |
|  |  | *11^MM^/11^MM^,tm-1/tm-1* | T_2_ azygous control |  |  |  |  |  | 4 | 11 |
|  | TM-186 | *11^MM^/11^MM^,Tm-1^OE^/Tm-1^OE^* | T_2_ transgenic |  |  | 1 | 4 | 3 | 4 | 3 |
|  |  | *11^MM^/11^MM^,tm-1/tm-1* | T_2_ azygous control |  |  |  | 1 | 1 | 2 | 11 |
