# Supplementary material for: Contribution of the tobamovirus resistance gene Tm-1 to control of tomato brown rugose fruit virus (ToBRFV) resistance in tomato
Source: PLoS Genet. 2025 May 23;21(5):e1011725. doi: 10.1371/journal.pgen.1011725 (PMC12140429; doi:10.1371/journal.pgen.1011725)
Supplement: S8 Table — (DOCX) [file pgen.1011725.s010.docx]

| **Line** | **Description** | **Type** | ***Tm-1* Transcription Fold** |
| --- | --- | --- | --- |
| VC554 | *11^VC554^/11^VC554^,Tm-1/Tm-1* | Not Transgenic control | 1 |
| VC554-*Tm-1*-AS-1 | *Tm-1* Anti-sense in VC554 | T_0_ Transgenic | 0.06 |
| VC554-*Tm-1*-AS-2 | *Tm-1* Anti-sense in VC554 | T_0_ Transgenic | 0.84 |
| VC554-*Tm-1*-AS-3 | *Tm-1* Anti-sense in VC554 | T_0_ Transgenic | 0.07 |
| VC554-*Tm-1*-AS-4 | *Tm-1* Anti-sense in VC554 | T_0_ Transgenic | 0.05 |
| VC554-*Tm-1*-AS-5 | *Tm-1* Anti-sense in VC554 | T_0_ Transgenic | 0.11 |

**S10 Table. *Tm-1* transcription fold across VC554-*Tm-1*-AS T_0_ transgenic plants.**
