# Supplementary material for: Contribution of the tobamovirus resistance gene Tm-1 to control of tomato brown rugose fruit virus (ToBRFV) resistance in tomato
Source: PLoS Genet. 2025 May 23;21(5):e1011725. doi: 10.1371/journal.pgen.1011725 (PMC12140429; doi:10.1371/journal.pgen.1011725)
Supplement: S9 Table — (DOCX) [file pgen.1011725.s011.docx]

**S11 Table. Disease Severity Index (DSI) metrics show** **the** **number of plants in each DSI in the experiment analyzing the reduction of *Tm-1* expression in the resistant genotype VC554.**

|  | **Line** | **Description** | **Type** | **No. of plants showing DSI of** | | | | | | |
| --- | --- | --- | --- | --- | --- | --- | --- | --- | --- | --- |
|  |  |  |  | **0** | **0.5** | **1** | **1.5** | **2** | **2.5** | **3** |
|  | TM-187 | *11^VC554^/11^VC554^, Tm-1^AS^/Tm-1^AS^* | T_2_ transgenic |  |  | 1 | 5 | 5 | 3 | 1 |
|  |  | *11^VC554^/11^VC554^, Tm-1/Tm-1* | T_2_ azygous control | 11 | 2 | 1 |  |  | 1 |  |
|  | TM-188 | *11^VC554^/11^VC554^, Tm-1^AS^/Tm-1^AS^* | T_2_ transgenic | 4 | 3 | 3 | 3 | 1 |  |  |
|  |  | *11^VC554^/11^VC554^, Tm-1/Tm-1* | T_2_ azygous control | 6 | 3 | 2 | 2 | 2 |  |  |
|  | TM-189 | *11^VC554^/11^VC554^, Tm-1^AS^/Tm-1^AS^* | T_2_ transgenic | 8 | 6 |  | 1 |  |  |  |
|  |  | *11^VC554^/11^VC554^, Tm-1/Tm-1* | T_2_ azygous control | 5 | 2 | 3 | 4 | 1 |  |  |
