# Supplementary material for: Contribution of the tobamovirus resistance gene Tm-1 to control of tomato brown rugose fruit virus (ToBRFV) resistance in tomato
Source: PLoS Genet. 2025 May 23;21(5):e1011725. doi: 10.1371/journal.pgen.1011725 (PMC12140429; doi:10.1371/journal.pgen.1011725)
Supplement: S10 Table — (DOCX) [file pgen.1011725.s012.docx]

**S12 Table. *Tm-1* 1^st^ and *Tm-1* 2^nd^ expression level in *Tm-1* harboring genotypes.**

| **Genotype** | **Description** | **N** | ***Tm-1* copy** | **Average**  ***Tm-1***  **expression** | **Fold *Tm-1* transcription** |
| --- | --- | --- | --- | --- | --- |
| LA2825 | *11^LA2825^/11^LA2825^,Tm-1/Tm-1* | 2 | *Tm-1* 1^st^ | 0.01905±0.01 | 1 |
| LA2825 | *11^LA2825^/11^LA2825^,Tm-1/Tm-1* | 2 | *Tm-1* 2^nd^ | 0.00005±0.0 | 0.002 |
| VC554 | *11^VC554^/11^VC554^,Tm-1/Tm-1* | 2 | *Tm-1* 1^st^ | 0.04346±0.03 | 1 |
| VC554 | *11^VC554^/11^VC554^,Tm-1/Tm-1* | 2 | *Tm-1* 2^nd^ | 0.0±0.0 | 0 |
| TM-35 | *F_3_(11^VC532^/11^VC532^,Tm-1/Tm-1)* | 2 | *Tm-1* 1^st^ | 0.07292±0.05 | 1 |
| TM-35 | *F_3_(11^VC532^/11^VC532^,Tm-1/Tm-1)* | 2 | *Tm-1* 2^nd^ | 0.00010±0.0 | 0.001 |
