# Supplementary material for: Contribution of the tobamovirus resistance gene Tm-1 to control of tomato brown rugose fruit virus (ToBRFV) resistance in tomato
Source: PLoS Genet. 2025 May 23;21(5):e1011725. doi: 10.1371/journal.pgen.1011725 (PMC12140429; doi:10.1371/journal.pgen.1011725)
Supplement: S11 Table — (DOCX) [file pgen.1011725.s013.docx]

| **Genotype** | **No. of plants showing DSI of** | | | | | | | **Average DSI** |
| --- | --- | --- | --- | --- | --- | --- | --- | --- |
|  | **0** | **0.5** | **1** | **1.5** | **2** | **2.5** | **3** |  |
| *11^VC53^/11^VC532^,tm-1^VC532^/tm-1^VC532^* | 18 | 1 | 1 |  |  | 1 | 1 | 0.3^A^ |
| *11^VC532^/11^VC532^,tm-1^VC532^/tm-1* | 27 | 1 |  |  | 1 |  | 1 | 0.2^A^ |
| *11^VC532^/11^VC532^,tm-1/tm-1* | 7 | 1 | 2 |  |  |  |  | 0.1^A^ |
| *11^VC532^/11^MM^,tm-1^VC532^/tm-1^VC532^* |  |  |  |  | 5 | 1 | 26 | 2.8^B^ |
| *11^VC532^/11^MM^,tm-1^VC532^/tm-1* | 1 |  |  |  | 9 | 2 | 47 | 2.8^B^ |
| *11^VC532^/11^MM^,tm-1/tm-1* |  | 1 |  |  | 2 | 2 | 18 | 2.7^B^ |
| *11^MM^/11^MM^,tm-1^VC532^/tm-1^VC532^* |  |  |  |  |  |  | 8 | 3^B^ |
| *11^MM^/11^MM^,tm-1^VC532^/tm-1* |  |  |  |  |  | 1 | 22 | 2.9^B^ |
| *11^MM^/11^MM^,tm-1/tm-1* |  |  |  |  | 1 |  | 12 | 3^B^ |

**S13 Table. Disease Severity Index (DSI) metrics show** **the** **number of plants in each DSI in the F_2_ population utilized** **for two way analysis to evaluate tm-1^VC532^ effect on symptom severity.**
