# Supplementary material for: Contribution of the tobamovirus resistance gene Tm-1 to control of tomato brown rugose fruit virus (ToBRFV) resistance in tomato
Source: PLoS Genet. 2025 May 23;21(5):e1011725. doi: 10.1371/journal.pgen.1011725 (PMC12140429; doi:10.1371/journal.pgen.1011725)
Supplement: Attachment — Submitted filename: Response to Reviewers.pdf [file pgen.1011725.s015.pdf]

Authors reply to comments

Reviewer #1:
The authors have found that a locus controlling tolerable to the tobamovirus tomato brown rugose fruit virus can work synergistically with a well known but relatively weak tobamovirus resistance gene Tm-1 to provide resistance to the virus. Although incremental in nature, the work may pint a way towards aiding in the control of the growing pandemic of tomato brown rugose fruit virus.

A: We thank the reviewer for the review.

Other comments
Title - name the virus in full. No reason why readers of a genetics journal will know what this is. Throughout the manuscript there is an inherent assumption that readers are already tobamovirus experts and a lack of clarity in exposition.

A: The title has been changed accordingly

Line 110. 'Levitzky et al. [21] also discovered... can spread ToBRFV' implies the bees transmitted the virus to other plants and acted as vectors. However, I dio not think that any evidnce for this was presnted - only that bees became contaminated with viable inouclum, which is not the same thing as acting as a vector.Please correct.

A: The sentence has been changed to – “Levitzky et al. [21] discovered that pollinating bumblebees (Bombus terrestris), can mechanically disseminate ToBRFV through buzz pollination, demonstrating its strong transmission potential”.

line 117. Please add dates of access for all URLs

A: Dates were added accordingly.

Data presentation is unsatisfactory, often unclear, and over-assumes background knowledge of the readership:
Table 1 Line 176 and elsewhere throughout the manuscript. With respect to a disease severity index, since this is a subjective measure, it would be helpful for the readership to be able to see examples 0 – 4 levels of symptoms (is it just appearance or stunting? etc.).

A: A new figure showing infected plants with different DSI was added to the manuscript, please see Figure 1.

Statistically, I do not think it is valid to have standard errors around subjective values, although I know it is often done, but is really not necessary. It would be more useful to know what proportion of plants in each group showed each DSI value. The description of the DSI values in materials and methods will not help anyone except an expert in tobamoviral infections of tomato (at line 180.... the line numbering in the ms is all over the

place). Again, try and consider who your audience is here.

A: (I) All the tables were amended accordingly, the DSI standard error was deleted.
(II) All the raw data was added as supplementary tables (see below), specifically the proportion of plants in each DSI level was added, please see S3, 5, 7-9, 11.
(III) Line numbering was corrected, sorry about that.

The raw data is not available as supp data: I would assume this is a requirement for publication. This applies to other tables in the manuscript. Similarly, just mentioning 'optical density' with saying that is ELISA (?) OD at what wavelength etc. does not help the clarity for readers who are not plant virologists (even then, some clarity is helpful since none of us are mind-readers). The authors are trying to publish in Pols Geneetics, not Annals of Applied Biology or Plant Disease and should tke this into account.

A: (I) All the raw data was added as supplementary tables, please see tables S3 to S13.
(II) The ELISA wavelength was added to the appropriate tables, and the ELISA protocol is described in Methods and Materials section.

Table 2 . Same comments about DSI as made re Table 1.What readers probably want to see is a good collection of representative images- there's no limit as to what you put in supplementary files. Why not use this opportunity to convince, persuade and impress the readers of the work's importance?

A: A DSI figure was added to the manuscript and Table 2 was corrected accordingly, as mentioned above.

Page 17. Table 3 not Tables 3. Again, the DSI with statistics is not helpful (and possibly not statistically valid - see earlier comments) , previously mentioned issues with ELISA data, and it is not clear how many plants were involved and therefore what the real statistical power was here. Table 4 etc similar points.

A: Tables 3 was changed to table 3. A DSI figure was added to the manuscript and Table 3 was corrected accordingly, as mentioned above.

Reviewer #2:
The manuscript presents an important and well-executed study on the genetic basis of resistance to tomato brown rugose fruit virus (ToBRFV), a pathogen of significant concern for tomato production worldwide. The research builds upon previous findings, providing compelling evidence of the interaction between the Tm-1 gene and a tolerance-conferring locus on chromosome 11. The experimental approach is well-designed, employing overexpression and RNAi-silencing strategies to dissect the role of Tm-1 in resistance. The results are clear and contribute valuable insights into the molecular mechanisms underlying resistance and tolerance to ToBRFV.The discussion effectively integrates the findings within the broader context of viral resistance in tomato, offering a logical

interpretation of the results and highlighting their potential applications in breeding programs. The manuscript is well-structured and clearly written, facilitating the comprehension of the study's implications.

A: We thank the reviewer for the review and the above summery.

Only minor formal issues should be addressed.
The authors should carefully review the use of upper/lower case and italics when referring to virus names, ensuring consistency with standard nomenclature.

A: Virus names were corrected accordingly.

Additionally, a thorough proofreading will help correct any minor typographical errors that may remain. These minor revisions should be easily addressed and will further improve the manuscript's clarity.Overall, this is a strong and relevant study that will be of great interest to researchers working on plant-virus interactions and resistance breeding.

A: The manuscript was proofread by an expert prior to submission.

Reviewer #3:
Authors (Zinger et al.) previously identified a locus on chromosome 11, in combination with a second locus mapped closely to the Tm-1 region on chromosome 2 from a tomato brown rugose fruit virus (ToBRFV)-resistant line (VC554). However, the Tm-1 locus was missing from the ToBRFV-tolerant line (VC532) but have the same locus on chromosome 11.In the present study, authors conducted various experiments to determine the role of Tm-1 for its contribution to ToBRFV resistance. In evaluating 60 F3 lines that were segregating for ToBRFV resistance, they confirmed again the association of the Tm-1 locus in combination with the chromosome 11 locus for ToBRFV resistance.Using transgenic tomato plants over-expressing the Tm-1 in the tolerance or the susceptible genotype background, they determined that the Tm-1 in combination with the chromosome 11 locus in the tolerance genotype achieved a high level of resistance to ToBRFV. However, the Tm-1 gene alone in the susceptible genotype yielded only some level of tolerance to ToBRFV without significant reduction in the virus titer.Silencing of the Tm-1 gene in the resistance genotype resulted a diverse response to ToBRFV infection. Although one plant with Tm-1 silencing, two others did not, these phenomena may be due to an incomplete silencing of the Tm-1 gene. Genome resequencing revealed the presence of two copies of the Tm-1 gene in the resistance genotype, but only one copy of the tm-1 gene in the tolerance genotype.Token together, these results offered strong evidence to support the contribution of the Tm-1, in combination with the locus in chromosome 11 resulting in the ToBRFV resistance.

A: We thank the reviewer for the review and the above summery.

However, following comments and suggestions would need to be considered in revisions.
1. All the data on (data not shown) need to be included in the complementary materials.

A: All the raw data was added as supplementary tables, please see tables S3 to S13.

2. All the table headings should be with one sentence only. The second sentence should be moved down to the footnote section.

A: All the table headings were changed accordingly.

3. Line 51. Tomato (Solanum lycopersicum) is… to Tomato (Solanum lycopersicum L.) is…

A: Tomato (Solanum lycopersicum) was changed to Tomato (Solanum lycopersicum L.)

4. Line 113. There are more countries than those listed here, it may be good to list EPPO for more comprehensive worldwide distribution (https://gd.eppo.int/taxon/tobrfv).

A: A reference to the EPPO was added.

5. Line 123. Missing a citation of Jaiswal et al., 2024, https://www.mdpi.com/2223-7747/13/5/5816.

A: The citation of Jaiswal et al., 2024 was added.

6. Page 15, on symptom severity, need to provide symptom photos in the supplementary materials. (the line number was not continuing from this page).

A: A new figure showing infected plants with different DSI was added to the manuscript, please see Figure 1.

7. Page 15, on the LA2825 (Tm-1/Tm-1 genotype in cv. Moneymaker), why the transgenic Tm-1 plants would yield milder symptoms than the LA2825 plants with homozygous for the Tm-1 gene?

A: Following this comment, a sentence was added to the discussion explaining the milder symptoms of the transgenic Tm-1 plants. Line 392, page 26 of the manuscript with track changes, "The elevated symptom severity observed in the line LA2825 harboring Tm-1 compared to the T_2 transgenic populations, can be attributed to the higher expression levels of Tm-1 in the transgenic plants relative to its normal expression levels in LA2825."

8. Page 26, line 77: change "we cloud not confirm" to "we could not confirm".

A: The sentence was corrected accordingly.

9. Page 26, line 83: change given to Given.

A: The sentence was corrected accordingly.

10. Page 29, line 155: What is the VC552 genotype? Need to explain this line was derived from a cross between VC532 and VC554.

A: There is no line VC552, this was a mistake. The correct line is VC532, and the page was corrected.

11. Page 41, Lines 380-381, the reference 12 is not complete.

A: Reference 12 was corrected
